# Baseline mapping of Lassa fever virology, epidemiology and vaccine research and development

**DOI:** 10.1038/s41541-018-0049-5

**Published:** 2018-03-20

**Authors:** Hoai J. Hallam, Steven Hallam, Sergio E. Rodriguez, Alan D. T. Barrett, David W. C. Beasley, Arlene Chua, Thomas G. Ksiazek, Gregg N. Milligan, Vaseeharan Sathiyamoorthy, Lisa M. Reece

**Affiliations:** 1 Department of Pathology, University of Texas Medical Branch, Galveston, TX USA; 2 Galveston National Laboratory, University of Texas Medical Branch, Galveston, TX USA; 3 Department of Microbiology and Immunology, University of Texas Medical Branch, Galveston, TX USA; 4 Sealy Institute for Vaccine Sciences, University of Texas Medical Branch, Galveston, TX USA; 5 World Health Organization Collaborating Center for Vaccine Research, Evaluation and Training on Emerging Infectious Diseases, University of Texas Medical Branch, Galveston, TX USA; 6 Office of Regulated Nonclinical Studies, University of Texas Medical Branch, Galveston, TX USA; 7 Department of Information, Evidence and Research, World Health Organization, Geneva, Switzerland; 8 Department of Pediatrics, Division of Vaccinology, University of Texas Medical Branch, Galveston, TX USA

## Abstract

Lassa fever (LF) is a zoonotic disease associated with acute and potentially fatal hemorrhagic illness caused by the Lassa virus (LASV), a member of the family *Arenaviridae*. It is generally assumed that a single infection with LASV will produce life-long protective immunity. This suggests that protective immunity induced by vaccination is an achievable goal and that cell-mediated immunity may play a more important role in protection, at least following natural infection. Seropositive individuals in endemic regions have been shown to have LASV-specific T cells recognizing epitopes for nucleocapsid protein (NP) and glycoprotein precursor (GPC), suggesting that these will be important vaccine immunogens. The role of neutralizing antibodies in protective immunity is still equivocal as recent studies suggest a role for neutralizing antibodies. There is extensive genetic heterogeneity among LASV strains that is of concern in the development of assays to detect and identify all four LASV lineages. Furthermore, the gene disparity may complicate the synthesis of effective vaccines that will provide protection across multiple lineages. Non-human primate models of LASV infection are considered the gold standard for recapitulation of human LF. The most promising vaccine candidates to date are the ML29 (a live attenuated reassortant of Mopeia and LASV), vesicular stomatitis virus (VSV) and vaccinia-vectored platforms based on their ability to induce protection following single doses, high rates of survival following challenge, and the use of live virus platforms. To date no LASV vaccine candidates have undergone clinical evaluation.

## Epidemiology of Lassa fever

Lassa fever (LF) is a zoonotic disease associated with acute and potentially fatal hemorrhagic illness caused by Lassa virus (LASV), a member of the *Arenaviridae* family. LF was first described and LASV was first isolated in 1969 when two missionary nurses became ill and died in the town of Lassa, Nigeria. Since then, LF has been shown to be prevalent in many West African countries, including Benin, Guinea, Liberia, Côte d’Ivoire, Mali, Nigeria, and Sierra Leone. However, the seroprevalence of LF varies within these endemic areas with reported incidence being highest in forested regions of West Africa.^1–3^ For example, in Sierra Leone, the prevalence of seropositive individuals varies from 8% in coastal regions to 52% in the Eastern Province^4^ whereas in Guinea, the reported seroprevalence varies from 4 to 55%.^1^ This is likely due to the fact that forested parts of endemic regions harbor large populations of the reservoir rodents (*Mastomys spp*.) capable of transmitting the virus to the human population. LF disease severity may be higher in Sierra Leone compared to Nigeria, and it has also been suggested that LASV has evolved to have increased virulence during its spread through West Africa. This is supported to some extent by molecular studies that have identified differences in genome abundance and translation efficiency for LASV strains from those areas.^5^ However, socioeconomic and human genetic factors also cannot be ruled out as possible sources of variations in LF disease severity.

Evidence of possible LASV endemicity has also been reported in Senegal, Burkina Faso, Ghana, Cameroon and Central African Republic.^2,6–8^ However, much of this surveillance was in part based on serological prevalence studies of rodents, for which seropositivity may have resulted from either LASV infection or possibly infections due to related arenaviruses. Since 1980, ten confirmed cases of LF have been imported to countries outside of Africa, and a recent imported case in Germany resulted in post-mortem infection of a mortuary worker.^9–12^

LF is mainly transmitted through contact with infected rodents (see Maintenance and Transmission of LASV section below) and, to a lesser extent, person-to-person contact. Therefore, those living in rural areas are generally at the greatest risk, especially in communities with poor sanitation and/or crowded living conditions. Healthcare workers caring for LF patients without proper personal protective equipment are also at risk, and nosocomial transmission has been estimated by modeling to comprise up to 20% of infections during some LF outbreaks.^13^ It is estimated that there are approximately 100,000–300,000 clinical infections in West Africa per year, with approximately 5000 deaths;^14–18^ however, the 2015 outbreak in Nigeria appears to have a case fatality rate of 50%.^19^ Peak incidence usually occurs around March, when the dry season transitions to a wet season,^7,20^ however, estimates of clinical disease case numbers are crude since the diagnosis and surveillance of LF cases are not uniformly performed. In addition, recent civil wars and other disturbances in hyperendemic regions of Africa have severely impeded LF surveillance and control.^15^ Overall, there has not been any analysis of economic burden attributed to LF, but given the estimated annual incidence of disease per year; it is likely to be significant in endemic areas of Africa. Finally, a better understanding of LF epidemiology will be needed to undertake clinical evaluation of vaccine candidates.

## Clinical LF disease

LF occurs in all age groups and both sexes and is associated with a broad spectrum of clinical manifestations. The incubation period following infection with LASV is usually between 7–10 days, with a maximum of 21 days. The clinical presentation of LF is mild or asymptomatic in the majority (80%) of infections.^7,20^ The onset of symptomatic disease is usually gradual, starting with mild fever, weakness, and general malaise. After a few days, headache, sore throat, muscle pain, chest pain, nausea, vomiting, diarrhea, cough, and abdominal pain may occur.^1,4,21,22^ In mild cases the fever subsides, and the patient usually recovers. Other cases progress towards a more severe illness. Symptoms include hemorrhage, respiratory distress, facial swelling, and fluid in the pulmonary cavity. Shock, seizures, tremor, disorientation, and coma have also been reported during this stage of the disease.^7,23,24^ Approximately 15–20% of hospitalized LF patients (roughly estimated at 1–3% of all cases) die from the illness,^7^ generally within 2 weeks after the onset of symptoms due to multi-organ complication and/or failure involving the liver, spleen or kidneys. Signs of acute kidney failure have been associated with fatal outcomes and hepatitis is frequent and moderately severe in patients diagnosed with LF.^25^ Pregnant women are more likely to have severe illness due to infection with LASV than women who are not pregnant,^4^ with maternal case fatality rates as high as 80% and nearly 100% mortality in fetuses. Infection in infants can result in “swollen baby syndrome” with edema, abdominal distension, bleeding and often death whereas symptoms in children (2 years of age and older) are similar to those seen in adults.^23,25^ Neurological problems have also been described in LF patients including hearing loss and encephalopathy. Various degrees of deafness, which may develop during both mild or severe cases,^24^ have been shown to occur in patients who survive the disease. Hearing partially returns after 1–3 months in approximately half of these cases while the remainder experience permanent loss of hearing.

Symptoms of LF are varied and non-specific, making clinical diagnosis often difficult (see additional discussion below in diagnostics section), especially early in the course of the disease. However, in survivors, a long-lasting production of antibodies occurs, and chronic infection is not established.

## Maintenance and transmission of LASV

The animal reservoir of LASV has been understood to be the multimammate rat, *Mastomys natalensis*, which is prevalent in most parts of West Africa. However, recently reported evidence suggests that other rodent species may also be hosts for LASV: the African wood mouse, *Hylomyscus pamfi* (Nigeria), and the Guinea mouse, *M. erythroleucus* (Nigeria and Guinea).^26^ Rodents infected with LASV do not display clinical signs of disease, but can shed the virus in their urine and feces throughout their entire life. Transmission between rodents is likely horizontal with some vertical transmission.^27^ These rodents breed frequently and produce large numbers of offspring, and are thus numerous in most regions of Africa. Transmission of LASV to humans is common, since these rodents scavenge on human food items and readily colonize areas where humans live. Multiple potential routes of virus transmission to humans have been described, including the gastrointestinal, and/or respiratory routes, and/or direct infection via abrasion of the skin. In terms of vaccine development, this may be important in terms of a vaccine inducing protective immunity against different routes of infection by the virus. Transmission to humans is assumed to occur through contact with virus-infected rodent excreta via eating rodent-contaminated food, exposure to contaminated objects, and inhalation of tiny particles in the air contaminated with virus-infected rodent excretions (urine or dried feces [although feces is often contaminated with urine]) and possibly dried rodent blood. In one study, it was shown that residences of patients with LF were 10 times more likely to be infested with rodents than residences with no reported cases.^28^ In addition, rodents are sometimes used as a food source and infection may occur when these rodents are caught and prepared for consumption.^24^ Individuals with a history of routine rodent consumption are more than twice as likely to have serological evidence of LASV infection when compared to individuals who do not consume rodents, and will also have a four-fold higher rate of deafness (one of the common neurological deficits) following LASV infection.^29^

Human-to-human transmission may also occur after exposure through direct contact with infected tissue, blood, secretions, or excretions of infected individuals.^22^ For example, the virus is excreted in urine for 3–9 weeks post infection and remains in semen for 3 months; however, the extent of sexual transmission is unknown.^20^ Epidemic spread of the disease via human-to-human transmission has not been widely observed, except for a couple of reports of nosocomial outbreaks, which were probably due to poor infection control practices (i.e., lack of appropriate personal protective equipment, use of contaminated items, failure to adequately disinfect between patients by handwashing) that facilitated transmission from patient-to-patient or to care givers. This includes contact with the corpse of a LF case.^12^ In addition, there is no evidence of airborne spread between humans.^24^

## LASV taxonomy and virology

LASV is a member of the family *Arenaviridae*, genus *Mammarenavirus*. Within this genus, LASV is categorized further as a member of the Old World arenaviruses based on serology, geography, and host distribution.^18,30^ This group includes related Lujo, Okahandja, Wenzhou, Lunk, Gairo, Mariental, Mobala, Ippy, Mopeia (MOPV), Merino Walk, Menekre, Gbagroube, Morogoro, Kodoko, Luna, and lymphocytic choriomeningitis (LCM) viruses.^31^ Additionally, LASV strains are grouped into four lineages based on genetic variation.^32^ The prototype strain is Josiah (lineage IV from Sierra Leone) and is utilized for most studies of LASV and vaccine candidates. Evaluation of recently isolated strains suggests the emergence of an additional fifth lineage (Cote d’Ivoire and Mali), although it has not been officially recognized.^3^ Lineages I-III have only been isolated in Nigeria, whereas lineage IV has been isolated in several West African countries. The complete genome sequences of several LASV strains are available as well as a considerable number of partial sequences for isolates from both humans and rodents. These analyses revealed a high level of sequence diversity among the four lineages of LASV, with up to 27 and 15% divergence at the nucleotide and amino acid (AA) levels, respectively.^32^ Table 1 shows AA identities for GPC and NP among the five different lineages^3,32^ since these two proteins are commonly targeted for vaccine epitopes. For the GPC, inter-lineage variation is between 5.1–8.4% at the AA level. In the NP, that variation is increased to 6.3–10.7%. Taking this heterogeneity among LASV strains into account will be critical for development of assays to detect and identify all LASV lineages, and for development of effective vaccines that will provide protection across multiple lineages.

**Table 1:** LASV AA Identities

	Lineage I-LP		Lineage II-NIG08-04		Lineage III-NIG08-A18		Lineage IV-Josiah		Lineage V*-AV	
	GPC	NP	GPC	NP	GPC	NP	GPC	NP	GPC	NP
Lineage I-LP	100%	100%								
Lineage II-NIG08-04	92.2%	90.9%	100%	100%						
Lineage III - NIG08-A18	92.2%	89.6%	93.9%	90.7%	100%	100%				
Lineage IV-Josiah	93.1%	90.5%	93.5%	89.6%	94.1%	91.6%	100%	100%		
Lineage V*-AV	91.6%	89.3%	92.1%	90.3%	93.7%	92.6%	94.9%	93.7%	100%	100%

*Proposed lineage

The LASV genome is composed of two segments of negative-sense RNA. Both segments encode open reading frames in an ambisense strategy. The large (L) segment is approximately 7.2 kilobases (kb) long and encodes for the viral RNA dependent RNA polymerase (RdRp) protein (termed L or LP) and the multifunctional Z matrix protein.^33^ The LP contains four proposed domains.^34^ Domain 1 harbors an endonuclease. Homologous structure has also been shown in the influenza polymerase acidic protein^35^ that facilitates the cleavage of host 5′ mRNA caps necessary for viral transcription. The third domain of LP contains conserved RdRp motifs. To date, domains 2 and 4 have no identified enzymatic or regulatory activity. The viral Z protein houses three domains: N terminal, Really Interesting New Gene (RING), and C terminal. The N terminal domain includes a myristoylation site allowing the protein to localize and embed into the cellular plasma membrane. The RING domain chelates Zn^2+^ ions and is critical to protein-protein interaction with LP and NP.^36–38^ Finally, the C-terminal domain contains conserved late-domains necessary for interaction with the host endosomal sorting complexes required for transport system, specifically protein Tsg-101.^39^ The small (S) segment is approximately 3.4 kb in length and encodes the NP and the GPC. The virion form of GPC is a trimer consisting of heterodimers, each containing glycoprotein 1 (GP1) and glycoprotein 2 (GP2), and stable signal peptide (SSP). LASV GPC is post-translationally cleaved by cellular Subtilisin Kexin Isozyme 1/Site 1 Protease (SKI-1-S1P).^40^ The SSP helps to localize the protein complex to the membrane. GP1 contains the receptor binding domain and GP2 is a type 1 fusion protein.^41^ After cleavage, all three proteins remain associated as a glycoprotein complex. Recently, the structure of the prefusion ectodomain of Lassa GP has been solved, indicating that low pH-driven conformational changes in both GP1 and GP2 occur, and that this structure is critical in binding to the cellular receptor.^42^

For LASV, the extracellular receptor is generally accepted to be matriglycan, which is a xylose-glucaronic acid sugar on α-dystroglycan, a cell surface glycoprotein interacting with the extracellular matrix and that GPC, rather than GP1 alone, binds to this molecule. Other receptor molecules have been identified^43^ and with the recent expansion of LASV isolates and lineages, there may be possibilities for alternative unknown cellular receptors. Following endocytosis and acidification of the endosome, the receptor binding domain is altered to allow the binding of lysosomal-associated membrane protein 1.^41^

## Animal models for LF

LASV infects several laboratory animal species including mice, guinea pigs, and non-human primates (NHPs). Typically, fully immune competent mouse (e.g., BALB/c) and guinea pig (e.g., Hartley) strains do not provide lethal models when studying LASV infection. Historically, the mouse models for LF have consisted of various immune knock out strains (STAT-1 KO, IFN-α/βR^−/−^) that develop lethal or clinical infections but are not ideally suitable to immune response studies.^44^ Recent studies have utilized CBA/J mice to evaluate the CD8^+^ response to certain vaccine candidates.^45^ Similarly, while outbred Hartley guinea pigs do not present with lethal disease to wild type Josiah LASV, the inbred Strain 13 guinea pig can be used as a lethal challenge model upon passage of the virus.^46,47^ Recently a lethal model of LASV infection of outbred guinea pigs for therapeutic antiviral testing was reported. Passage of Josiah strain LASV four times in Hartley guinea pigs resulted in a virus preparation with an approximate LD_50_ of 10^3^ TCID_50_. Intraperitoneal inoculation with 10^4^ TCID_50_ (10xLD_50_) resulted in a uniformly lethal outcome with an average time to lethal outcome of 15 days. The development and widespread use of this model is significant for vaccine and antiviral testing as it would allow use of commercially available outbred guinea pigs instead of reliance on the limited availability of inbred Strain 13 guinea pigs from private breeding colonies.^48^ Table 2 summarizes treatments under research and development for LF as studied in LASV small animal models.

**Table 2:** Treatments under research and development for Lassa fever evaluated in small animal models

Treatment	Efficacy [survival in %]	Animal model	Citation
Stampidine (Nucleoside analog)	^a^75% (25 mg/kg) and 90% (50 mg/kg)	CBA mice	141
Zidampidine (Nucleoside analog)	^a^25% (25 mg/kg)	CBA mice	142
Interferon-α **(**Interferon alfacon-1)	100% (10 mg/kg/d x 5 d); Synergistic effect with ribavirin to reduce mortality, FDA approved	Hamsters (Pichinde virus model)	143
Favipiravir (T-705)	100% (300 mg/kg/d x 15 d)	Guinea pigs, transgenic mice	48,116,144–146
Antibody therapy (Monoclonal)	^b^100% with a cocktail of five human monoclonals (30 mg/kg)	Guinea pigs	72
ST-193 (Small-molecule arenavirus entry inhibitor)	^c^62.5% (25 mg/kg and 80 mg/kg)	Guinea pigs	135,147

^a^Initial Stampidine or Zidampidine dosages were delivered 24 (h) and 1 h prior to LASV inoculation, followed by subsequent dosages at 24, 48, 72, and 96 h post inoculation

^b^Antibody cocktails were delivered at challenge (Day 0), followed by Days 3 and 6 post infection

^c^ST-193 was delivered 1 h prior to LASV inoculation, followed by daily treatments for a total of 14 doses

NHP models of LASV infection are considered the gold standard for recapitulation of human LF. Specifically, rhesus and cynomolgus macaques (*Macaca mulatta* and *M. fascicularis*, respectively) have been shown to express the hallmarks of LF: unchecked viremia, elevated liver enzymes, low proinflammatory cytokines (IL-1β, TNF-α, IL-8, IP-10), and low levels of T cell activation.^44,49,50^ Additionally, in the macaque model, high IL-6 production correlates with lethal outcome, which has also been shown in human patients.^51^ For this reason, NHP models are indispensable in studying the safety and efficacy of LASV vaccine candidates. Current vaccine candidates have been tested for immunogenicity and protection in mouse, guinea pig, and NHP models. An important caveat in vaccine testing using these models is that LASV is a rodent-borne virus and the immune systems of rodents may respond to and clear the virus differently compared to primates. For example, a recombinant vaccinia virus expressing the nucleoprotein of LASV protected guinea pigs from challenge with the Josiah strain^52^ whereas a similar LASV nucleoprotein-expressing vaccinia virus was only weakly protective in rhesus and cynomolgus monkeys.^15^

Gene transcription profiling studies in LASV-infected NHPs and human cells have provided important insights into the nature of LASV infection and may ultimately be instrumental in the development of biomarkers for disease severity or outcome. Quantitative RT-PCR analysis of PBMC mRNA from strain AV (lineage V) LASV–infected cynomolgus monkeys detected early activation of Type I interferon genes in monkeys that survived infection compared to only late-stage detection of these transcripts in nonsurvivors.^50^ Along with development of strong T cell and monocyte activation in survivors, these data suggested that early, vigorous activation of immune responses resulted in control of LASV infection. Later studies utilizing whole genome microarray analysis of PBMCs from Josiah strain (lineage IV) LASV-infected cynomolgus macaques revealed early induction of interferon-responsive genes, Toll-like receptor signaling pathways and lack of pro-inflammatory cytokine response genes.^53^ Further utilization of this type of mRNA profiling identified different transcriptional signatures in PBMCs from LASV and Marburg virus infected NHPs (including differences in expression of genes for heat shock proteins, immunoglobulins, cell adhesion molecules, the translational repressor SAMD4A and the tyrosine kinase TNK2) suggesting that transcriptional analysis might allow the ability to distinguish among pathogens at early stages of infection.^54^ Transcriptional profiling has also been performed using human PBMCs infected with either the Josiah strain of LASV or a vaccine candidate, the live attenuated reassortant of MOPV and LASV, ML29. In this model of the viremic stage of LASV infection, heightened expression of interferon-stimulated genes, genes regulating apoptosis, genes involved in the NF-κB pathway and coagulation pathway genes was detected in LASV-infected PBMCs compared to ML29-infected cells suggesting the possibility that biomarkers may be identified to predict disease outcome.^55^ It is tempting to speculate that genetic profiling may be helpful towards identifying immune biomarkers of protection for rational vaccine development. However, application of putative immune biomarkers identified from virus infection studies for prediction of correlates of vaccine-mediated protection may not be straightforward.

## Immune responses to LASV infection

The initial targets of infection for LASV are thought to be macrophages and dendritic cells (DCs).^56–58^ Following infection, LASV interferes with complete maturation and activation of these innate immune cells. Presentation of viral antigens by immature DCs may result in the development of tolerance rather than activation of adaptive immune cells.^59^ Consistent with this notion, infection of human DCs with related, but non-pathogenic, MOPV resulted in maturation of these antigen presenting cells and the ability to induce robust T cell responses in vitro, while infection with LASV resulted in diminished DC maturation and a limited in vitro T cell response.^57^ Despite this disruption of antigen presentation by LASV, both CD4^+^ and CD8^+^ T cell responses are detected during early infection from individuals who ultimately resolve the infection. Seropositive individuals in endemic regions have been shown to have LASV-specific CD4^+^ memory T cells recognizing epitopes in NP and GPC^60,61^ that are maintained for years after the infection. Preliminary T cell adoptive transfer studies have been performed to establish the protective efficacy of LASV-specific T cells. In these experiments, T cells from mice immunized with LASV GPC in a LCMV backbone did not clear LASV infection in recipient mice.^62^ However, this may have been partially due to interference in development of the LASV GPC-specific response by the extremely strong CTL (cytotoxic T lymphocyte) response to the LCMV NP. In support of a putative role for T cells in protection, a vaccine that elicited only weak antibody response, induced significant protection against lethal outcome in NHP.^15^ It is important to note that there is evidence of secondary infection (seroconversions), with no evidence of disease in individuals who have had a primary LASV infection. Further, testing by IFAT (immunofluorescence antibody test) shows that antibody wanes, but these individuals again have increased antibody levels, ostensibly because of reinfection or a boost.^14^ Thus, protective immunity induced by vaccination is considered an achievable goal.

The B cell response appears limited during the acute phase of LASV infection and higher antibody titers against LASV antigens are generally not detected until convalescence. In human LF patients, IgM and IgG antibodies have been detected with specificity to NP and GPC.^63–65^ In addition, mouse monoclonal antibody (mab) epitope mapping studies have identified several antibody targets in the viral NP, GP1 and GP2^66,67^ of Josiah strain using IFA, radioimmunoprecipitation assay, and competition ELISA. Neutralizing activity (50% plaque reduction) was detected for 7 of 17 GP1- and GP2-specific mabs tested in those studies. Recently, a study of neutralizing human mabs derived from LF survivors showed that the human mabs bind to quaternary epitopes of the prefusion GPC, and not GP1 or GP2 individually^68^ and the structure of the prefusion ectodomain of GPC bound to a human mab has been solved.^42^ The structural data indicates that the mechanism of neutralization derives from the ability of these antibodies to prevent the conformational changes required for GPC that are necessary for binding receptor(s) and for viral fusion with membranes.

Early studies of LF patient sera examined two measures of antibody titer: log_10_ neutralization index (LNI) and IFA.^69^ These studies showed that LNI titers were detected late after convalescence whereas IFA titers could be detected earlier. Passive transfer of LASV-immune antibodies from human or animal immune sera has been shown to provide protection against lethal infection in NHPs.^47,69^ A passive transfer study performed in guinea pigs suggested the degree of protection and in vivo suppression of LASV was closely associated with neutralization activity (via plaque reduction neutralization testing (PRNT) and LNI) and not antibody binding (ELISA) titers.^69^ Additionally, passive transfer of the late serum (high LNI) protected guinea pigs whereas early serum (low LNI) did not. Human mab cocktails have also been shown to provide protection in outbred guinea pigs when administered directly after challenge and were shown to have high binding affinities to the GPC of LASV lineages II, III, and IV.^70–72^ Although passive transfer of neutralizing antibodies has been shown to provide protection against lethal outcome in animal models, passive transfer of immune plasma in a controlled human trial did not confer protection.^73^ However, the neutralizing titer of the sera used in that trial was not known, and therefore, the potential of neutralizing antibodies for treatment of LF remains a possibility. Additionally, virus clearance does not necessarily correlate with high antibody as determined by IFA,^74^ and resolution of LASV infection in humans with undetectable LASV-specific neutralizing antibodies suggests a less important role for antibody in viral clearance during LF disease. Taken together, given the lack of correlation between development of neutralizing antibody titers with either the resolution of human LF disease or protection of vaccinated NHPs from lethality, there is a general consensus that cell-mediated immune responses are important for both virus clearance during LF and protection. However, it should be noted that recent work also established a role of antibodies in protection from infection.^42,68,72^

## Diagnostics

Clinical recognition of LF can be challenging due to the similar symptoms of other febrile illnesses prevalent to geographic regions containing LASV, such as other viral hemorrhagic fevers, malaria, shigellosis, and typhoid.^14^ Reliable laboratory diagnostics are critical for LASV confirmation, initiation of healthcare barrier precautions, patient isolation, treatment options, and contact tracing epidemiology for outbreak response. Currently, LASV diagnostics have relied on using detection of viral antigens, nucleic acids, and IgM/IgG antibodies. Many research grade enzyme-linked immunosorbent serologic assays (ELISA), reverse transcription polymerase chain reaction (RT-PCR), and tissue culture methods have been developed for identification of LASV infections. Lastly, postmortem diagnosis may be established through immunohistochemistry from formalin-fixed tissues.

RT-PCR is a rapid molecular tool for detection of LASV RNA in blood, tissues, and secretions during the early (acute) stages of infection and has been adapted for mobile laboratory field settings.^16,75–79^ Although RT-PCR is the prototypical “gold standard” for viral identification, this may not be adequate as the sole detection method given the high genetic diversity among LASV isolates, as such ELISA and/or tissue culture assays are recommended for secondary confirmation.^79^ Due to genetic diversity at the nucleotide level (up to 27% variability among all sequenced LASV isolates), primer sets may be designed for clinical labs to geographically reflect isolates circulating regionally.^32^ Improved primers for conventional RT-PCR that target highly conserved regions of the S- and L-segments and work on multiple lineages are available.^80,81^ Validated real-time RT-PCR assays detecting a broad range of LASV lineages and strains have not yet been reported. Additionally, oligonucleotide array hybridization coupling, nested-PCR techniques, LAMP, and resequencing and transcriptional profiling microarray chips are being examined for bolstering LASV detection methods and developing prognostic indicators.^54,82–86^ Many of these ‘in development’ approaches are research tools and will still be dependent on robust matching of target sequences and test sequences to maintain sensitivity and specificity.

Diagnosis of LF may also be established based on ELISA, which can detect IgM and IgG antibodies, or by identifying LASV antigens via an antigen capture assay.^24^ ELISA platforms have demonstrated robust use in the field without the need for expensive equipment. There are several research grade ELISAs having high sensitivity and specificity when cross-validated by other research methods, such as RT-PCR and IFA.^87–91^ Many research grade ELISA reagents have been developed, such as those utilizing sera from LASV exposed mice, rabbits, guinea pigs and NHPs. These have been used in various field research and diagnostic settings using indirect, and capture ELISAs for both antigen detection and serological analysis.^87–90,92,93^ There are commercially available ELISA kits developed for detection of “pan” LASV NP (from lineages II, III, and IV) antigen or IgM/IgG, though performance characteristics (sensitivity and specificity) have not been established for either kit, and field/clinical evaluations are underway (ReLASV® Pan-Lassa Antigen ELISA test kit (cat. 10003) and ReLASV® Pan-Lassa IgG/IgM ELISA test kit (cat. 10004, Zalgen Labs, Germantown, MD, USA, http://www.zalgenlabs.com/hemorrhagic-fever-tests-list-2.html). Additionally, there are commercially developed immune ascitic fluids, mabs to NP, GP1, and GP2, and antigen preparations, such as irradiated whole virus and recombinantly expressed NP (based on Josiah strain [lineage IV]), which can be adapted and used for antigen coating in indirect or capture sandwich ELISAs (Polyclonal Anti-Lassa Virus Hyperimmune Mouse Ascitic Fluid, cat. NR-48962; Lassa Virus, Josiah, Gamma-Irradiated, cat. NR-31822, BEI Resources, Manassas, VA, USA). Rapid antigen detection tests are also under development in endemic regions of Africa, such as a “dip-stick” method using lateral flow immunoassays (which tests for the presence of LASV NP from clades II, III, and IV) for use during acute stage LASV infection. One such platform (specific for lineage IV only) has been validated with sensitivity and specificity of 85 and 98.7% compared to RT-PCR, respectively, and has been approved as an in vitro diagnostic by the European Union CE Marking (ReLASV^®^ Antigen Rapid Test, Zalgen Labs, USA).^94^ These platforms may serve as cost-effective, presumptive tests (necessitating confirmation by other LASV diagnostic standards), which require only a small (<30 μL) blood specimen and quick (15 min.) diagnostic read-out.^75,88,94^

Potential ELISA cross-reactivity among antigenically similar Old World arenaviruses has not been carefully assessed, though Lujo, Mopeia, Mobala, Ippy and LCM-like viruses are antigenically related to LASV.^44,95–97^ Mobala and Ippy have documented serological cross-reactivity (via IFA) when tested with human convalescent serum from LF patients, as well as research grade LASV mabs.^97^ Furthermore, there is a possible limitation of these assays with regards to false-positive testing acute diagnosis of patients who present with LF-like etiologies but who may not have LASV infection. Patients, who develop a less-severe form of LF and survive, will convalesce with an IgG response to LASV antigens. Therefore, serological diagnosis of acute LF cases requires a demonstration of detectable IgM or significant increase in IgG titers in paired serum samples, or necessitates confirmation by other methods, such as RT-PCR or antigen detection and IgM ELISAs.^14,32,92,98–100^ Clearly, there is a need for development of standardized ELISA reagents for antigen and serological testing across lineages with established performance characteristics. Further development and standardization of immunoassays to support continuing improvements in surveillance for LF and for use in evaluation of candidate LF vaccines, would be enhanced by availability of well characterized reference panels comprising antisera for LASV and for related arenaviruses expected to circulate in LASV-endemic areas. However, it is recognized that finding samples of individuals who have been infected with other arenaviruses will be very difficult.

Isolation of LASV in cell culture remains a diagnostic method that can be employed during the acute phases of the disease,^88^ however, virus isolation has the limitations of requiring a week or more of culturing in high-containment laboratories of biosafety level 4 by trained personnel.^54^ Virus isolated in cell culture may be identified by immunoassays, such as PRNT, IFA, and western blotting, or RT-PCR, or virus sequencing can be accomplished.^101–103^

## Prevention and treatment of LF

Currently, there are no licensed vaccines for prevention or treatment of LF, thus measures to reduce risk of infection from multimammate rats are promoted, including storing food in rodent-proof containers, disposing garbage away from the community or home, and maintaining a clean living environment. As mentioned previously, contact with both the rats and their fecal material must be avoided to discourage spread of disease. The use of rat traps around the home as well as disposing of dead rodent carcasses using plastic bags can also facilitate avoidance of contact with possible infected rats.

To avoid human-to-human transmission, early recognition of human infection combined with patient isolation is necessary within a hospital setting. If a quarantined patient is determined to be infected, all healthcare workers in contact with said patient should wear personal protective equipment for viral hemorrhagic fever.^104^

Overall, LASV infection poses a significant healthcare burden in those living in rural areas where communities have poor sanitation and/or crowded living conditions and preventive measures include avoiding *Mastomys* rodents and minimizing risk of person-to-person transmission altogether.

### Therapeutics

Current treatment options for LF are limited and outcomes of said treatments are largely dependent on the patient’s presentation or phase of disease. Symptom management includes analgesics such as paracetamol, however, non-steroidal anti-inflammatory drugs and aspirin should be avoided due to increased risk of bleeding. If bleeding does occur, thrombocytopenia is corrected with platelet transfusions. Coagulation deficits can be treated with blood products such as fresh frozen plasma, while blood transfusions are reserved for those patients who are anemic with ongoing bleeding. Diarrhea occurs in approximately 50% of cases, and those with significant diarrhea should have electrolyte replacement as needed. Intravenous fluids should be started to maintain adequate fluidic volume in hospitalized patients.^104^

Ribavirin, a guanosine analog and broad spectrum antiviral drug, has been shown to improve outcome in both NHP animal models and in a clinical trial involving LF patients in Sierra Leone. When administered early during the course of infection, ribavirin has been shown to reduce viral load and improve patient outcomes when used in conjunction with palliative care, such as fluid and electrolyte balance, maintaining blood pressures, proper oxygen saturation of blood, and appropriate triage of secondary complications. IV administration of ribavirin involves a 2 g loading dose and 1 g every 6 h for 4 days, followed by 0.5 g given every 8 h for an additional 6 days^73^ whereas the oral loading dose is 35 mg/kg followed by 15 mg/kg three times a day for 10 days is currently recommended for post-exposure prophylaxis following a “high-risk” exposure to LASV.^105^ This drug is used “off-label” and widely accepted by many countries for the treatment of LF.^106,107^ It has additionally been sanctioned by the WHO in the model list of essential medicines for treatment of certain hemorrhagic fevers, including LF, and as a contingency investigational drug undergoing phase two clinical trials in the United States as a generic treatment for imported viral hemorrhagic fevers. Given the increased severity of disease and higher mortality of pregnant mothers infected with LF,^73,79,108,109^ treatment with ribavirin may be considered despite the contraindications of ribavirin for nursing and pregnant mothers due to teratogenic effects that have been observed with administration of ribavirin in animals.^110^ In those instances, physicians must weigh the risk-benefit to pregnant or nursing mothers with LF on a case-by-case basis.

However, there are limited clinical data demonstrating the efficacy of ribavirin treatment for LF. In one clinical study, the efficacy of oral or intravenous (IV) administered ribavirin was comparable in patients who had serum aspartate aminotransferase (AST) levels ≥ 150 U/L at the time of hospital admission, though the beneficial effect on outcome was higher if the drug was administered early during the course of LF.^73^ Treatment of patients with AST levels < 150 U/L had a CFR of 12% after treatment with oral ribavirin, which was not significantly different from untreated patients, perhaps due to the small sample size. Since individuals with lower AST levels normally have less mortality, the drug may not have had as profound an effect in reducing mortality as described for more severe cases. Early ribavirin treatment would most likely help in all cases, but in groups with lower mortalities, discerning effectiveness was not as easily achieved. Side effects of ribavirin include hemolytic anemia and infusion-related reactions such as rigors. When ribavirin is used as a post-exposure prophylactic, the adverse effects at the effective dose may be severe and can lead to poor treatment compliance.^29,104^ Given the relative lack of data on efficacy of ribavirin, it seems advisable to re-evaluate the therapeutic potential of ribavirin in all LF patients as stand-alone medication and potentially in combination with other experimental drugs.

Convalescent plasma containing high titers of neutralizing antibodies has also been evaluated with promising results in animal models such as guinea pigs and NHPs, and human mab cocktails with high binding affinity to GPCs of LASV lineages II, III, and IV have been shown to provide protection in outbred guinea pigs when administered directly after challenge.^70–72^ Very recently, a cocktail of human mab were shown to protect NHPs against LASV challenge even if given up to 8 days post virus challenge. Significantly, the antibodies in the cocktail recognize epitopes on the LASV-GP complex and bind GP from clades I-IV, leading to the conclusion that these antibodies may have potential for a cross-protective therapeutic for LASV in West Africa.^17^

In New World arenaviruses, such as Junín virus, convalescent sera have demonstrated efficacy in reducing mortality among Argentine hemorrhagic fever patients.^111,112^ However, there are mixed results in human LF cases treated with convalescent plasma (~3–4 ml/kg), and it has been suggested geographic matching of convalescent plasma, as well as titers of neutralizing antibodies per therapeutic dose, may be significant factors in effective passive therapy.^69,73,113,114^ A guinea pig animal model has indicated the degree of protection and in vivo suppression of LASV may be closely associated with neutralization ability (via PRNT and LNI).^69,70^ Additionally, there are concerns that the level of neutralizing activity in convalescent plasma collected from donors might be time-dependent (≥6 months post-infection required for induction of robust neutralizing antibody titers), and that unselective convalescent plasma may need to be concentrated to be therapeutically useful.^69^ Criteria for selection of human convalescent plasma are clearly needed if plasma is to be used therapeutically. Several other treatment options under research and development have yet to be deployed in clinical use and are outlined in Table 2. The broad spectrum antiviral drug favipiravir (T-705) and cocktails of neutralizing mabs currently appear to be promising candidates for further pre-clinical and clinical development. Favipiravir is an existing drug that has already undergone phase I and II trials for influenza virus infection.^115^ In addition, it has been clinically evaluated during the Ebola virus disease (EVD) epidemic in West Africa and has shown some—though not statistically significant—efficacy in EVD patients with low virus load on admission.^116^

### Vaccine candidates

A large number of candidate vaccines have been developed that have undergone preclinical evaluation in mouse, guinea pig, and non-human primate models (see Table 3) but to date, no LASV vaccine candidates have undergone clinical evaluation. Various strategies and platforms have been explored for candidate vaccines against LASV. However, the majority of vaccine researchers use antigens derived from the Josiah strain and assess protection via homologous challenge with Josiah virus. Early attempts at preclinical vaccine studies utilized irradiation-inactivated whole virus. Immunized test animals developed antibody responses against NP and GPC but the immunization regimen did not provide protection against lethal challenge.^117^ This implies the necessity of a cell mediated response in addition to an appropriate antibody response to the viral NP and GPC. Live attenuated or live-vectored vaccines express either LASV NP and/or GPC proteins and are favored because they are thought to induce a higher CD8^+^ T cell response. Currently, development of antigen specific CD8^+^ T cells has been shown in CBA/J mice and NHPs vaccinated with the live attenuated ML29 (L RNA segment from MOPV and S RNA segment from LASV)^118^ candidate and in NHPs vaccinated with the live VSV-vectored candidate.^119^ Alphavirus and *Salmonella* vectored vaccines showed induction of cell mediated immunity although these were not further characterized as CD8 positive or negative.^120–122^ All other candidates rely on (neutralizing) antibody concentrations/detection as a parameter of immunogenicity (Table 3). Other strategies utilize related Old World arenavirus (MOPV),^123^ Old World reassortants (MOPV/LASV, ML29),^122,124^ DNA platforms,^125,126^ inactivated virus,^118^ and live vectored recombinant viruses based on yellow fever 17D,^127^ alphavirus (Venezuelan equine encephalitis),^128,129^ VSV (rVSV),^18,130^ vaccinia (New York Board of Health (NYBH) and Lister strains) (rVACC).^123,131^

**Table 3:** Lassa virus vaccine candidate platforms in preclinical development

LASV candidate	Test species	Antigen/virus strain	No. of doses	Dose	Time to challenge	Survival	Test parameter	Date	Ref.
Recombinant vesicular stomatitis virus (rVSV)	Guinea pig strain 13	GPC/Strain Josiah	1	10^6^ PFU	28 d	100%	Antibody	2015	130
	Cynomolgus monkey	GPC/Strain Josiah	1	2-6 × 10^7^ PFU	28 d	100%	Antibody and T cell IFN-γ	2015 2005	18,130
DNA	Guinea pig strain 13	GPC/Strain Josiah	3	100 µg	63 d	100%	Antibody	2013	125
Venezuelan equine encephalitis (VEE)-like replicon	Mouse CBA/J	GPC/Strain Josiah	2	Unknown	Unknown	100%	T cell IFN- γ	2012	128
	Guinea pig strain 13	NP or GPC/Strain Josiah	3	10^7^ IU	84 d	100%	Antibody	2001	129
Recombinant yellow fever 17D	Guinea pig strain 13	GPC/Strain AV	1	10^5^ PFU	21 d	80%	Antibody	2006	132
	Guinea pig strain 13	GP1 & GP2/Strain Josiah	2	5 × 10^6^ PFU	44 d	83%	Antibody	2011	127
	Marmoset	GPC/Strain Josiah	2	Unknown	30 d	0%	Unknown	2012	45
Recombinant mopeia/Lassa virus (ML29)	Guinea pig strain 13	GPC & NP/Strain Josiah	1	10^3^ PFU	30 d	100% (60–100% protection with simultaneous replication of ML29 and LASV Strain Josiah)	Antibody	2007 2005	122,124
	Marmoset	GPC & NP/Strain Josiah	1	10^3^ PFU	30 d	100%	Antibody and T cell IFN-γ	2008	124
Salmonella vectored LASV-NP	BALB/c mice	NP/ Strain unknown	2	5 × 10^9^ CFU	25 d	37%	Antibody and CTL activity	2001, 2000	120,121
Mopeia virus	Rhesus monkey	Whole Virus Strain/ Unknown	1	10^4^ PFU	37 d	100%	Antibody	1998	123
Vaccinia (Lister) vectored virus	Guinea pig Hartley	NP/Strain GA391	1	10^7^ PFU	28 d	100%	Unknown	1987	131
Vaccinia (NYBH) vectored virus	Rhesus monkey	GPC/Unknown	1	10^9^ PFU	28-37 d	100%	Antibody	1998	123
	Rhesus and cynomolgus monkeys	GP1, GP2, GPC, NP, GPC/NP/ Strain Josiah	1 or 2	10^9^ PFU	62-488 d	89% GPC 90% GPC/NP	Antibody	2000	123
Inactivated virus	Rhesus monkey	Whole Virus Strain/ Unknown	6	Unknown	108 d	0%	Antibody	1992	117
Nanocarrier with recombinant GP1 (envelope glycoprotein) encapsulated into polymersomes	C57BL/6 mice	GP1/Unknown	2	10 μg	14 and 28 d	0% (all animals sacrificed at 28 d; no survival data)	Antibody/CD4 T cell/B cell	2017	133
Modified vaccinia Ankara-virus like particle (MVA-VLP)	Mouse species not declared	Not declared	1	10^3^ PFU	10 d	100%	T cell	2017	134

The most promising candidates to date are the ML29, plus the VSV and vaccinia-vectored platforms based on induction of protection following a single dose, high rate of survival following challenge, and the use of live virus platforms.^45^ For example, Safronetz et al., undertook a study where guinea pigs were immunized with 10^6^ PFU of the live-attenuated vaccine candidate rVSV-LASVGP via IP injection (not an appropriate route for humans), and then challenged with 10^4^ TCID_50_ Josiah strain. Guinea pigs did not show any clinical signs of disease and were protected against genotype IV LASV and divergent clade I LASV strain Pinneo from Nigeria; however, immunization did not prevent LASV infection (i.e., no sterilizing immunity).^130^ ML29 vaccination of strain 13 guinea pigs was also shown to protect against both lineage IV and lineage II viruses.^128^ In comparison, challenge experiments for all other candidates in guinea pigs were performed using the well-characterized lineage IV Josiah strain only.^18,118,122,125,127,129–132^ Recently, Galan-Navarro et al. has used nanotechnology to develop a recombinant GP1 immunogen encapsulated into polymersomes (PS) as nanocarriers. This nano-complex promotes intracellular MHCII loading. C57BL/6 mice immunized with adjuvanted PS (LASV GP1) by the intradermal (ID) route into the 4 footpads, showed superior humoral responses with enhanced frequencies of LASV-specific B cells (but not free antigen), indicating a distinct marked effect of PS-delivery on the antibody response. This nanocarrier vaccine induced the formation of antibodies with high binding affinity to LASV GP1, increased levels of CD4 T cells, and IgG-secreting B-cells.^133^ In a recent report, GeoVax Labs, Inc. has announced the results of efficacy testing in a mouse model using a LASV recombinant vaccine based on their modified vaccinia Ankara-VLP platform. A single dose of GEO-LM01 (GeoVax Labs, Inc., Smyrna, Georgia, USA) delivered IM conferred 100% protection after challenge with a lethal dose of virus (1000 PFU). Vaccinated mice produced a strong T cell immune response at 10 days post infection (DPI).^134^ At the time of writing, this study has not appeared in the peer-reviewed literature.

While studies with small animal models such as mice and guinea pigs generate important results, critical data are obtained using non-human primate models. One macaque model of lethal LASV was utilized to assess the protective efficacy of LASV-GP-neutralizing antibodies on the basis of four lineages. It appears that a relatively high or low dose of challenge virus elicited immunogenicity in macaques. In one report, 3,500 PFU LASV-GP was an effective immunization dose,^17^ in contrast, only 1000 PFU LASV (Josiah) delivered subcutaneously was effective in another study.^135^ In an additional study, cynomolgus macaques were vaccinated IM with 2 × 10^7^ PFU of a rVSV expressing the LASV GP (VSVΔG/LVGPC); although this is a high dose, similar high doses have been used for rVSV-Ebola vaccine candidates with an acceptable safety profile. After vaccination, none of the NHPs showed clinical signs, indicating that the rVSV was tolerated well. On 28 days post immunization, the animals were challenged IM with 10^4^ PFU LASV Josiah strain. None of the VSVΔG/LVGPC-vaccinated monkeys showed clinical signs of illness and all were fully protected. Further, no viremia or vaccine vector shedding was detected after vaccination (prior to virus challenge) in the macaques. By the day of the LASV challenge, all vaccinated primates had developed moderate-to-high-level IgG antibody titers as well as low-level neutralizing antibody titers against LASV. All vaccinated macaques were fully protected against the high LASV challenge dose indicating rVSVΔG/LVGPC is a potent stimulator of humoral and cellular immunity.^18^ In a similar study using rhesus macaques, protective efficacy of a vaccinia recombinant expressing LASV-GPC was confirmed. Animals were vaccinated with 10^9^ PFU LASV-GPC by the ID route and on day 37 post immunization were challenged with LASV (Josiah, 10^4^ PFU subcutaneously). All animals survived the challenge. On days 7–9 post challenge, low transient viremia at 10^2^–10^3^ PFU/ml was detected, and animals developed a mild fever with some abnormalities, including depressed platelet function, neutrophilia, and lymphopenia. Only 2 of 4 primates developed antibodies to LASV GP. In a broader investigation, using recombinant vaccinia expressing different regions of LASV GPC and NP, it was determined that both GP1 and GP2 are required for protection, and full length GPC is necessary to protect the vaccinated animals against LASV challenge.^45,123^

In a recent DNA vaccine study, cynomolgus macaques received 100 μl ID-EP (ID electroporated) administration of 2.5 mg LASV-GPC DNA. Three DNA vaccinations at 3-week intervals were followed by a single LASV challenge (Josiah strain, 1000 PFU delivered IM) 4 weeks after the last vaccination. The NHPs not only survived, but did not exhibit viremia or fever at any time point post challenge. Neutralizing antibody levels increased in the vaccinated animals, peaked around 21 DPI and then slightly declined at the end of the study.^126^

A reassortant vaccine platform, MOPV/LASV (clone ML29) was designed to retain the non-pathogenic profile of MOPV while keeping the desired induction of strong protective immunological responses against LASV. This clone has the genotypic characteristics of the L-segment RNA of MOPV (strain An20410) and the S-segment RNA of LASV Josiah strain. This chimeric live-attenuated vaccine was found to be safe, immunogenic and efficacious in marmosets (*Callithrix jacchus*). Animals were inoculated subcutaneously with 10^3^ (low dose) and 10^6^ (high dose) PFU ML29. At 30 DPI, the NHPs were challenged with LASV (Josiah) at 10^3^ PFU by the subcutaneous route. The ML29 immunization increased CD14 + lymphocytes as well as increasing the levels of CD3 + T cells. There was also over-expression of HLA-DR, P, Q, and recruitment of CD3 + cells to the hepatic parenchyma in all animals immunized with the high dose of ML29. On day 28 in marmosets immunized with ML29 high dose, the average number of TNF-α-secreting cells was 7-fold higher than in those animals vaccinated with ML29 low dose. The data suggest that LASV immunity was conferred through cellular responses.^45,124,136^

Although there is a relatively low level of HIV-1 seroprevalence in the young adult population within LASV endemic regions (e.g., approximately 1% in Sierra Leone), it poses a potential safety concern for live LASV vaccine candidates. Accordingly, a study was designed to determine if ML29 was safe as well as immunogenic in macaques during advanced stages of SIV infection. ML29 (10^3^ PFU delivered subcutaneously) was tested in simian immunodeficiency virus (SIV)-infected rhesus monkeys, and none of the macaques developed chronic infection or clinical signs of disease. Furthermore, the ML29 vaccine did not induce viremia in the healthy controls, while low titer transient viremia was detected in SIV-infected rapid and median progressors, and biodistribution data of solid organ tissues (spleen, liver, kidney, heart, lung, and adrenal glands) were negative for the presence of ML29. All vaccinated monkeys developed ML29-specific cell-mediated and humoral responses.^33^

Yellow Fever 17D has been investigated as a vaccine vector for the expression of LASV antigens. A single dose of recombinant virus, YF17D/LASVΔGPC at 10^5^–10^6^ PFU was used to inoculate marmosets. Booster immunizations were performed on days 14 or 30 followed by LASV challenge on day 30 with 1000 PFU LASV (Josiah). This immunization regimen did not induce any protective immune response and all vaccinated animals died with LF clinical signs.^45^ An additional investigation involved the cloning of a SIVMAC239 Gag construct encoding E and NS1 viral proteins yielding the recombinant virus, YF17D/SIVGag_45-269_. This recombinant virus was able to induce SIV-specific CD8 + T cells in rhesus macaques.^45^

Finally, Mopeia virus is immunologically related to LASV. MOPV shares NP and GP2 epitopes with LASV, but it lacks epitopes responsible for eliciting full protection against LASV.^123^ The recombinant LASV GPC vaccinia virus, rVACC-LSGPC,^52,137^ was tested in rhesus macaques using MOPV as the control. Fisher-Hoch et al. tested eight rhesus macaques where two monkeys received 10^9^ PFU of NYBH strain vaccinia virus, and four were given the same dose of rVACC-LSGPC. The two remaining animals were vaccinated with 10^4^ PFU MOPV 37 days prior to challenge. All animals were vaccinated ID in four sites (each forearm and lateral upper legs). Viral challenge consisted of administering 10^4^ PFU of LASV (Josiah) subcutaneously in all but two of the monkeys. The last two NHPs were challenged 284 days post immunization with the same virus dose but delivered by the IM route. The two monkeys receiving NYBH vaccinia virus died 12 and 15 days post challenge. The two monkeys vaccinated with MOPV and the four that received rVACC-LSGPC prior to viral challenge all survived but all six animals exhibited a mild fever with transient, low-titer Lassa viremia.^123,137^ In contrast, animals who received MOPV virus withstood LASV challenge with little or no signs of illness. However, the macaques immunized with MOPV and rVACC-LSGPC developed antibodies to LASV GP1 and GP2 prior to challenge, with additional antibody induced to LASV NP. Monkeys vaccinated with only rVACC-LSGPC, exhibited an increase in antibody levels against these glycoproteins post challenge. NHPs vaccinated with Mopeia and NYBH viruses did not produce any GP1/GP2 antibodies.

One important consideration in vaccine development will be the potential risk of presumably immune-mediated neurological complications (hearing deficit) noted in natural LASV infection.^24,138,139^ Safety studies addressing this concern will need to be conducted on all clinical candidates.

A WHO Target Product Profile had recently been developed for LF and this will aid in the development of candidate vaccines to proceed to clinical evaluation.^140^

## Conclusions

To date no candidate vaccine has advanced to clinical evaluation. A path forward to future licensure of vaccines and therapeutics for LASV may rely on a traditional approval pathway involving multi-site efficacy trials in endemic regions of West Africa. Indeed, the apparent annual incidence of disease would support this route rather than utilizing the US Food and Drug Administration’s “Animal Rule.” However, such a strategy will necessitate the development of standardized assays and more robust regional surveillance to effectively quantify the burden of disease and establish suitable sites for clinical trials, and to aid in the definition of suitable efficacy endpoints. In particular, knowledge on geographical spread of the virus as well as incidence of clinical disease and subclinical infections in the various endemic areas is insufficient. These activities would all require investments into clinical and diagnostic infrastructure in endemic countries but would not be prohibitive to successful trials. Selection of an efficacy trial endpoint based on preventing clinical disease or infection in general will require reliable diagnostic tools capable of providing confirmed diagnosis of LASV infection, including, but not limited to, real-time RT-PCR or antigen detection assays covering all LASV lineages and serological assays to detect and discriminate natural infection and vaccine response. As noted above, many development studies and surveillance programs have used different immunological measurements including ELISA antibody levels, neutralizing antibody levels, or viral titers. There is a demonstrated need for standardized reagents across LASV lineages with established and repeatable performance characteristics. Studies in animal models show that guinea pigs are a suitable small animal model to evaluate candidate vaccines, but additional studies are needed in NHPs for advanced nonclinical developed prior to undertaking clinical evaluation. The nonclinical studies have identified roles for both neutralizing antibodies and cell mediated immunity in protection against disease caused by LASV following infection or vaccination, but variability in outcomes in different animal models and with different immunization strategies suggests that identification of a specific immunological endpoint that correlates with protection may be challenging. Both total and neutralizing antibody titers and LASV-specific T cell responses will likely need to be measured during clinical testing. Finally, at the present time, the most promising vaccine candidates are live attenuated recombinant MOPV/LASV reassortant and live recombinant VSV and vaccinia-vectored vaccines.
